# The bioprospecting potential of insect venoms as antibiotics: a mini review

**DOI:** 10.3389/fmicb.2025.1729786

**Published:** 2025-12-12

**Authors:** Henrique G. Riva, Angela R. Amarillo-S

**Affiliations:** 1 Wild Animal Conservation Institute (ICAS), Campo Grande, MS, Brazil; 2 Independent Researcher, Bogotá, Colombia

**Keywords:** Hymenoptera, antimicrobial peptides, biofilm, mastoparan, venom-derived compounds, bioprospecting

## Abstract

The global rise of antimicrobial resistance has intensified the search for new antibiotic candidates from unconventional biological sources. Insect venoms, although underexplored compared to other venomous taxa, harbor a chemically diverse array of antimicrobial peptides (AMPs) with significant therapeutic promises. This mini review synthesizes evidence from 15 original studies published over the past 15 years that examined the antimicrobial potential of insect venom components. Most investigations have focused on Hymenoptera—wasps, bees, and ants—where peptides such as mastoparans, polydim-I, macropin, melectin, and panurgines that exhibit broad-spectrum activity against multidrug-resistant bacteria while maintaining low toxicity toward mammalian cells. Collectively, these findings highlight insect venoms as a promising resource for antibiotic discovery. Nevertheless, critical challenges remain regarding peptide stability, delivery, pharmacokinetics, and clinical validation. Addressing these gaps through integrative approaches combining molecular, computational, and translational research will be key to advancing insect venom peptides as next-generation anti-infective agents.

## 1 Introduction

Antibiotics have revolutionized medicine and contributed to a rapid rise in life expectancy alongside other advances in human health as vaccines, anesthesia and image exam technologies. On the other side, an arms race began with antibiotic-resistant infections that is estimated to have killed 4.95 million people worldwide just in 2019 (Murray et al., 2022; Guan et al., 2025; Ganavi and Ramesh, 2024). In this context, finding new safe antimicrobial peptides have an increasing importance and insects may present an opportunity due to their extreme resistance to bacterial infections (Wu et al., 2018; Oñate-Garzón et al., 2016). Bioprospecting research on reptile venoms is extensive; however, insect venoms are rarely investigated (Pineda et al., 2001; Arsanios et al., 2020). Insects thrive well in microbe-rich environments and rely on their cellular and humoral immune systems for defense. The humoral system, among other constituents, uses antimicrobial peptides (AMPs), molecules that are vital for combating pathogenic microorganisms, including bacteria, fungi, and viruses.

Considering published peer-reviewed articles, a few insect groups rise having the most potential when exploring venom bioprospecting as antibiotics. A strong limitation of the landscape literature however is the high focus on the order Hymenoptera (ants, bees and wasps). The order seems to attract more attention from researchers, probably due to a highly developed venom apparatus producing toxins that sometimes could also affect humans or maybe because of complex social structures that bring more interest. The venom of other orders like Lepidoptera, Coleoptera, Hemiptera or Diptera was left aside on the search for antibiotic peptides.

Among the insect venoms, Hymenopteran insects represent a rich repository of an underexplored source of bioactive antimicrobial peptides with significant therapeutic potential (Konno et al., 2019; Agarwal et al., 2022; Ganavi and Ramesh, 2024). Therefore, it’s an untaped opportunity to explore new drugs derived from or inspired by proteins from insects (Abella et al., 1999; Pineda et al., 2001). Mastoparan peptides from the wasp *Eumenes micado*, ponericins from the predatory ant *Pachycondyla goeldii*, and melectin from the bee *Melecta albifrons*, are all examples of venom-derived molecules with antimicrobial properties (Cabrera et al., 2019; Konno et al., 2019; Ko et al., 2020; Orivel et al., 2001). Research with Lepidoptera’s venom did not show bioprospecting potential for antibiotics (Gritti et al., 2023), however the hemolymph of venomous *Lonomia obliqua* could be a source of antimicrobial molecules (Nascimento et al., 2016; Hayashida et al., 2019; Riva and Amarillo-S, 2023). Research with the venom of other insect groups is even more scarce, with only one example published almost 20 years ago found in this review of potentially useful molecules in the hemipteran order with the venom of the assassin bugs *Rhynocoris marginatus* and *Catamirus brevipennis*, that use their salivary venom to paralyze their prey (Sahayaraj et al., 2006).

Social wasps and bees have proven to be valuable reservoirs of antimicrobial molecules. For instance, polydim-I, a peptide from the Neotropical social wasp *Polybia dimorpha*, demonstrated potent antimycobacterial effects *in vitro* and *in vivo*, significantly reducing bacterial loads of *Mycobacterium abscessus* in infected mice without cytotoxicity to mammalian cells (das Neves et al., 2016). In bees, peptides such as macropin from *Macropis fulvipes* have shown potent antimicrobial and anti-biofilm properties while sparing mammalian cells from toxicity. These peptides exert their effects by binding to bacterial membrane components like lipopolysaccharides and peptidoglycan, disrupting membrane integrity, and in some cases, synergizing with conventional antibiotics (Ko et al., 2017; Ko et al., 2020).

Considering the scarce research on insect venoms as sources for antibiotic molecules and the unexplored potential of this group, this mini review aims to explore and highlight peer-reviewed articles published in the last 15 years that investigate the bioprospecting potential of components of insect venoms for therapeutic use against bacteria.

## 2 Search strategy

This mini-review obtained data from a search conducted in English across the PubMed, Scopus, Directory of Open Access Journals, Google Scholar and Web of Science databases to identify peer-reviewed studies investigating the antimicrobial potential of insect venoms. Searches were performed using variations of the following search function: (“venom-derived” OR venom) AND (antibiotic OR antimicrobial) AND (insect OR bee OR wasp OR bug* OR honeybee OR ant). The inclusion criteria encompassed original experimental studies published in the last 15 years (between January 2010 and October 2025) that examined peptides or other bioactive components derived from insect venoms with demonstrated antibacterial activity. The determined period criteria is a limitation of this mini review, considering that a deep and critical meta-analysis is not the focus of this document that rather emphasizes an overview on the subject. Articles regarding hemolymph, whole body or other non-venom components of insects were excluded. Review articles, conference abstracts, and studies focused on non-insect arthropods (as spiders or scorpions) were discarded as well. Articles were selected by a single reviewer initially based on the title and abstract. Three additional articles were found in secondary references.

## 3 Insect venom characteristics and applicability

### 3.1 Overview of published articles on insect venoms

All the articles included in this mini review (Table 1) focused on species belonging to the order Hymenoptera, confirming this group as the principal source of insect venoms investigated for antimicrobial potential. A total of 15 peer-reviewed studies from five countries examined venoms from 13 insect species, comprising one ant (Menk et al., 2023), five bees (Čujová et al., 2013; Kim et al., 2013; Ko et al., 2017; Ko et al., 2020; Park et al., 2018; Jeon et al., 2024), and seven wasps (das Neves et al., 2016; Ha et al., 2017; Rangel et al., 2017; Ganavi and Ramesh, 2024; Konno et al., 2019; Silva et al., 2017; Silva et al., 2020).

**Table 1:** List of studies of insect venom molecules with antibiotic potential.

References	Molecule(s) studied	Tested microorganisms	Animal tests	Insect species
Čujová et al. (2013)	Panurgines (novel peptides)	Effective against: *Bacillus subtilis*, *Escherichia coli*, *Micrococcus luteus*, *Staphylococcus aureus*, *Pseudomonas aeruginosa* and the yeast *Candida albicans*	No animal infection model reported	Solitary bee, *Panurgus calcaratus*
das Neves et al. (2016)	Polydim-I (mastoparan)	*Mycobacterium abscessus*; ESKAPE pathogens (*Enterococcus faecium*, *S. aureus*, *Klebsiella pneumonia*, *Acinobacter baumanii*, *P. aeruginosa* and *Enterobacter* sp.) & multidrug-resistant bactéria (MDR) clinical isolates	*In vivo*: mice infected models (reduced bacterial loads); low mammalian toxicity	Social wasp *Polybia dimorpha*
Rangel et al. (2017)		ESKAPE pathogens and MDR	No animal infection model reported. *In vitro* determination of minimum inhibitory concentration using ELISA	Social wasp *Polybia dimorpha*
Ganavi and Ramesh (2024)	Crude venom and AMPs	*S. aureus* and *E. coli*	No animal model, spot-on-lawn assay method of antimicrobial analysis	Social wasps: *Ropalidia marginata* and *Vespa tropica*
Silva et al. (2017)	Polybia-MPII (mastoparan)	*S. aureus*; *M. abscessus*; *C. albicans*; *C. neoformans*	Murine topical *S. aureus* infection model (reduced load)	Social wasp, *Pseudopolybia vespiceps*
Silva et al. (2020)	Mast-MO (engineered from mastoparan-L)	Multiple pathogens incl. ESKAPE bacteria	*In vivo*: mouse sepsis & skin infection models (efficacy + immunomodulation)	Social wasp *Polybia paulista* (engineered peptide)
Ha et al. (2017)	Mastoparan V1 (MP-V1)	*Salmonella* serotypes (*Gallinarum*, *Typhimurium*, *Enteritidis*)	Recombinant production via *E. coli*; no mammal model	Social wasp *Vespa velutina*
Kim et al. (2013)	Kazal-type serine protease inhibitor (AcKTSPI)	Effective against: *B. subtilis* and *Bacillus thuringiensis* And has antifungal activity against *Beauveria bassiana*, *Fusarium graminearum* Not effective against: *E. coli*	No animal models, liquid growth inhibition assay was used for antimicrobial analysis	Asian honeybee, *Apis cerana*
Park et al. (2018)	Vitellogenin (AcVg)	Effective against: *E. coli*, *B. thuringiensis*, and the fungus *B. bassiana*	No animal model, microbial binding assays for antimicrobial analysis	Asian honeybee, *Apis cerana*
Konno et al. (2019)	EMP-EM1/EMP-EM2 (eumenine mastoparans)	Effective against: *S. aureus*, *Staphylococccus saprophyticus*, *Staphylococcus epidermis*, *B. Subtilis*, *E. coli*, *Escherichia cloacae*; yeast *C. albicans* Not effective against: *B. thuringiensis*, *Proteus miriabilis*, *P. aeruginosa*	No mammal models; *in vitro* hemolysis & mast cell assays	Solitary wasp, *Eumenes micado*
Ko et al. (2017)	Macropin	Broad-spectrum bacteria including drug-resistant strains; biofilms	No large-animal model; low cytotoxicity & hemolysis	Solitary bee, *Macropis fulvipes*
Ko et al. (2020)	Melectin	Effective broad-spectrum against: *S. aureus*, *P. aeruginosa*, *Salmonella typhimurium*, *K. pneumoniae*, *E. coli*	No systemic animal model; low cytotoxicity	Solitary bee, *Melecta albifrons*
Jeon et al. (2024)	Osmin	Effective antimicrobial and anti-biofilm activity against drug resistent *K. pneumoniae*	*In vivo*: mice infected models	Solitary bee, *Osmia rufa*
Menk et al. (2023)	Predicted AMP candidates (*Odontomachus chelifer* transcriptome)	No specific bacteria were analyzed	No animal model, computational prediction of AMPs candidates	Tropical trap-jaw ant *Odontomachus chelifer*
Monincová et al. (2014)	Macropin (MAC-1)	*P. aeruginosa*, *B. subtilis*, *E. coli*, *M. luteus*, *S. aureus*	No animal model, drop-diffusion test for antimicrobial analysis	Solitary bee, *Macropis fulvipes*

South Korea accounted for the largest share of publications (six), followed by Brazil (five), Czech Republic (two) and other countries with one article: India, and Japan. Most investigations were conducted within universities (14 out of 15 studies), while only one originated from a research institute (Rangel et al., 2017). Among these, four studies employed animal models to assess *in vivo* antimicrobial activity (das Neves et al., 2016; Silva et al., 2017; Silva et al., 2020; Jeon et al., 2024), and none reported clinical trials in humans.

### 3.2 Diversity of insect venom AMPs

The antimicrobial peptide (AMP) repertoire of insect venoms is taxonomically diverse, with most known examples described from Hymenoptera, including wasps, bees, and ants. Venoms from solitary and social wasps contain families such as mastoparans, anoplin, and polydim-I, which exhibit strong antimicrobial activities (Cabrera et al., 2019; Ganavi and Ramesh, 2024; das Neves et al., 2016; Silva et al., 2017; Silva et al., 2020). Ant venoms contribute ponericins, a heterogeneous group divided into subfamilies (G, W, and L) with distinct antibacterial spectra (Orivel et al., 2001; Menk et al., 2023). In bees, notable examples include osmin from *Osmia rufa*, melectin from *Melecta albifrons*, and macropin from *Macropis fulvipes*, all of which exhibit potent antibacterial activity, with melectin and macropin additionally showing low cytotoxicity toward mammalian cells (Jeon et al., 2024; Ko et al., 2017; Ko et al., 2020). A list of references of studies of Hymenoptera venom molecules with antibiotic potential can be found below (Table 1).

Non-Hymenopteran AMPs are less documented, but Hemipteran reduviid bugs (*Rhynocoris marginatus*, *Catamirus brevipennis*) produce venom with antibacterial activity against several human pathogens (Sahayaraj et al., 2006), however, this article was not included in Table 1 because it was published more than 15 years ago. Computational mining and transcriptomic studies have predicted additional cecropin-like and defensin-like peptides across other insect taxa, underscoring a largely untapped diversity (Guan et al., 2025; Menk et al., 2023).

### 3.3 Biological and ecological function

Venom AMPs often serve a dual function: disabling prey and preventing microbial contamination. In predatory ants such as *Pachycondyla goeldii*, ponericins likely reduce pathogen load from prey carcasses introduced into the nest (Orivel et al., 2001). Social wasps, frequently exposed to environmental microbes, may use venom AMPs such as polydim-I and mastoparans for colony-level pathogen defense (das Neves et al., 2016; Ganavi and Ramesh, 2024). For reduviid bugs, antimicrobial venom factors may protect the predator from opportunistic infections acquired during feeding on immunocompromised prey (Sahayaraj et al., 2006; Yan and Adams, 1998).

Evolutionary pressures from both prey–predator interactions and communal living could have selected for peptides with potent and broad antimicrobial activity (Ascoët et al., 2023; Cabrera et al., 2019).

### 3.4 Structural and physicochemical characteristics

Most venom-derived AMPs are short (10–35 amino acids), linear, cationic, and amphipathic, often adopting an *α*-helical structure in membrane-mimicking environments (Cabrera et al., 2019; Konno et al., 2019; Ko et al., 2020). These structural features enable insertion into and disruption of bacterial membranes. Net positive charge facilitates electrostatic attraction to negatively charged microbial surfaces, while hydrophobic faces promote membrane penetration. Variations in hydrophobicity and helix stability correlate with differences in antimicrobial potency and cell selectivity (Ko et al., 2017; Ko et al., 2020). Some peptides, such as polydim-I, also show stability in the presence of physiological salt concentrations, a desirable pharmacological trait (das Neves et al., 2016; Rangel et al., 2017).

### 3.5 Mechanisms of action

Venom AMPs primarily target bacterial membranes through mechanisms such as pore formation, membrane thinning, and detergent-like disruption (Cabrera et al., 2019; Konno et al., 2019; Ko et al., 2017). Electrostatic binding to lipopolysaccharide (LPS) in Gram-negative bacteria or peptidoglycan in Gram-positive bacteria initiates the interaction (Ko et al., 2020; Oñate-Garzón et al., 2016). Certain peptides, including macropin, not only disrupt cell membranes but also inhibit biofilm formation and eradicate established biofilms of drug-resistant pathogens (Ko et al., 2017). Synergy with conventional antibiotics has been documented, enhancing bacterial clearance at lower doses (Ko et al., 2020). Differences in lipid composition between Gram-positive and Gram-negative membranes influence peptide susceptibility, explaining variable spectra within a single AMP family (Orivel et al., 2001; Menk et al., 2023).

### 3.6 Spectrum of antimicrobial activity

AMPs from insect venoms display a broad range of activity against Gram-positive and Gram-negative bacteria, including multidrug-resistant (MDR) strains such as *Staphylococcus aureus*, *Pseudomonas aeruginosa* and *Escherichia coli* (Ko et al., 2017; Ko et al., 2020). Some, like polydim-I, show potent antimycobacterial activity *in vitro* and *in vivo* against *Mycobacterium abscessus* (das Neves et al., 2016; Rangel et al., 2017). Although less common, certain venom peptides also exhibit antifungal (Oñate-Garzón et al., 2016; Čujová et al., 2013; Kim et al., 2013) and antiparasitic activity (Konno et al., 2019). Spectrum differences can be significant even within a peptide family—for example, ponericin subfamilies differ in Gram-type specificity and potency (Orivel et al., 2001).

### 3.7 *In vivo* efficacy and toxicity

*In vivo* studies demonstrate that insect venom AMPs can effectively reduce bacterial burdens without significant host toxicity. Polydim-I significantly reduced lung, spleen and liver bacterial loads in mice infected with *M. abscessus* by provoking cell wall disruption without exhibiting cytotoxicity toward mammalian cells (das Neves et al., 2016). Effectiveness *in vivo* of another mastoparan peptide, Polybia-MPII, was reported by Silva et al. (2017) in topical treatment of skin infections of *S. aureus* in mice, reducing bacterial load and promoting wound healing. Additionally, the synthetic peptide mast-MO was tested in skin and intraperitoneal infections in mice with enhanced antimicrobial activity by destabilizing the bacterial outer membrane and exhibited immunomodulatory properties by increasing leukocyte migration to the infection site and repressing proinflammatory factors (Silva et al., 2020). Although the mastoparan family was discovered decades ago, it has been poorly evaluated using *in vivo* tests (Silva et al., 2017).

Hemolysis assays generally reveal low to moderate activity, with some peptides, like melectin and macropin, showing strong selectivity for bacterial over mammalian cells (Ko et al., 2017; Ko et al., 2020). Nonetheless, peptides such as certain mastoparans exhibit dose-dependent hemolysis, highlighting the importance of balancing efficacy and cytotoxicity (Cabrera et al., 2019; Silva et al., 2017).

Results of the peptide osmin are also interesting by significantly reducing *K. pneumoniae* bacterial burden and pro-inflammatory cytokine expressions in infected mouse model (Jeon et al., 2024).

### 3.8 Synthetic production and optimization

Advances in peptide synthesis and recombinant expression have facilitated the production of native and analog insect venom AMPs. Solid-phase peptide synthesis (SPPS) has been used to create both wild-type sequences and modified variants with improved activity profiles (Cabrera et al., 2019; Silva et al., 2020). Recombinant systems, including *E. coli* secretion pathways, enable cost-effective production of peptides like mastoparan V1 (Ha et al., 2017). Optimized analogs have demonstrated improved antimicrobial potency against MDR bacteria and, in some cases, reduced hemolysis (Silva et al., 2020; Rangel et al., 2017).

## 4 Bioprospecting future and knowledge gaps

The last decade has witnessed remarkable progress in the discovery of AMPs from insect venoms, positioning them as promising candidates for next-generation antibiotics. The 15 original studies examined in this review demonstrate that insect venoms contain a chemically diverse arsenal of peptides, many with potent activity against multidrug-resistant (MDR) pathogens. These investigations, ranging from the isolation of novel peptides to *in vivo* validation, provide an experimental foundation for developing insect venom-derived antimicrobials. Still, significant knowledge gaps and translational challenges remain.

### 4.1 Expanding the molecular repertoire

Research has so far identified a limited but diverse set of insect venom AMPs, including mastoparans from social wasps (Silva et al., 2017; das Neves et al., 2016; Ha et al., 2017; Konno et al., 2019; Cabrera et al., 2019; Rangel et al., 2017), melectin (Ko et al., 2020), macropin (Monincová et al., 2014) and panurgines (Čujová et al., 2013) from solitary bees, and Kazal-type inhibitors (Kim et al., 2013) from honeybees. Venomic and transcriptomic approaches have expanded the scope, identifying putative peptide arsenals in ants such as *Odontomachus chelifer* (Menk et al., 2023) and *Tetramorium bicarinatum* (Ascoët et al., 2023). Despite this progress, insect venom AMPs remain underexplored compared to other venomous taxa, and the true chemical diversity is likely far greater. Future bioprospecting efforts should emphasize deep sequencing and integrative venomics in unexplored insect lineages, coupled with functional screening.

### 4.2 Toward clinical translation

Only a few venom peptides have progressed to *in vivo* validation. Notably, mastoparan-derived peptides and engineered variants demonstrated efficacy in murine models of lethal bacterial infection (Silva et al., 2020). Polydim-I was also effective against mycobacteria infections and multi-resistant strains, it also showed to be safe to mice on *in vivo* assays and against mammal cells *in vitro* (das Neves et al., 2016; Rangel et al., 2017). These successes represent critical translational milestones. However, most other insect venom peptides remain at the *in vitro* stage, with limited pharmacokinetic, stability, or immunogenicity assessments. Systematic pre-clinical pipelines, including toxicity profiling and formulation strategies, are urgently needed to move the field closer to clinical trials.

Venom peptides are inherently unstable in physiological environments, where proteolysis rapidly degrades them. To date, a few studies have addressed delivery challenges beyond demonstrating activity in buffered systems. Some analogues, such as mastoparan derivatives, have been modified for improved stability and immunomodulation (Silva et al., 2020), but further exploration of delivery systems—nanoparticles, liposomes, and peptidomimetics—remains essential for clinical translation.

### 4.3 Broadening the therapeutic scope

While most studies focus on bactericidal properties, several insect venom AMPs also display antifungal (das Neves et al., 2016), antibiofilm (Rangel et al., 2017), or immunomodulatory effects (Silva et al., 2020). This multifunctionality may allow development of dual-action therapeutics that both kill pathogens and enhance host responses. Moreover, selective immunomodulation, as demonstrated by Silva et al. (2020), could mitigate harmful inflammation in infections. Future studies should explore this broader therapeutic landscape, as it could differentiate insect venom peptides from other AMP sources.

### 4.4 Knowledge gaps and research priorities

Despite the promise of those 15 studies, several gaps persist:

Taxonomic bias: Most characterized peptides come from wasps and bees, with ants only recently explored (Ascoët et al., 2023; Menk et al., 2023). Vast insect lineages remain chemically unexplored. Lepidoptera, Coleoptera, Hemiptera and Diptera are orders known to include species that produce venom, however, no peptides were described from those insects. It’s possible that other insects lack the necessity for highly evolved venom, however, it seems more probable that Hymenoptera is just more charismatic, considering only one study was found in this mini-review that specifically states that an insect’s venom (Lepidoptera) has no bioprospecting potential as an antibiotic (Gritti et al., 2023).
Incomplete mechanistic data: While many peptides are assumed to act via membrane disruption, few studies provide detailed molecular or structural analyses.
Translational bottlenecks: *In vivo* testing remains scarce (Silva et al., 2020; Guan et al., 2025), and there is limited understanding of pharmacokinetics and toxicity.
Resistance potential: Unlike conventional antibiotics, AMPs are thought to limit resistance, but systematic long-term studies confirming this in insect venom AMPs are lacking.
Clinical pipeline integration: No insect venom peptide has advanced to human trials, and integration into pharmaceutical pipelines is still in its infancy.

## 5 Conclusion

Insect venoms are increasingly recognized as a rich source of AMPs with therapeutic potential. The studies revised here highlight how bioprospecting—guided by venomics, computational design, and rational engineering—can yield peptides capable of overcoming multidrug resistance and even curing lethal infections *in vivo* (Silva et al., 2020; Guan et al., 2025). To unlock this potential, future research must address stability, delivery, and toxicity challenges, while expanding discovery efforts into neglected insect lineages. Multidisciplinary integration of evolutionary biology, structural biochemistry, and drug development will be crucial. AMPs that already show promising results *in vivo*, like polydim-I (das Neves et al., 2016), Polybia-MPII (Silva et al., 2017), osmin (Jeon et al., 2024) or mast-MO (Silva et al., 2020), require further studies to evaluate reproductivity of results, security in other species and effectiveness with other bacteria to reach the stage of human trials. Synthetic peptides and chemical modification could be important strategies for addressing stability and cytotoxicity issues before clinical application, as exemplified by the peptide mast-MO (Silva et al., 2020).

In conclusion, while only 15 original studies published in the last 15 years were found on this mini-review regarding bioprospecting with insect venom AMPs as antibiotics, they collectively provide compelling proof-of-concept. The field is promising, where addressing key knowledge gaps could transform these natural toxins into lifesaving therapeutics.
